# Supplementary material for: TFAM organizes DNA into compact higher order structures
Source: bioRxiv. 2026 Apr 27:2026.04.24.720727. Preprint. [Version 1] doi: 10.64898/2026.04.24.720727 (PMC13142342; doi:10.64898/2026.04.24.720727)
Supplement: Supplement 1 [file NIHPP2026.04.24.720727v1-supplement-1.pdf]

bioRxiv preprint doi: https://doi.org/10.64898/2026.04.24.720727; this version posted April 27, 2026. The copyright holder for this preprint (which was not certified by peer review) is the author/funder, who has granted bioRxiv a license to display the preprint in perpetuity. It is made available under aCC-BY 4.0 International license.

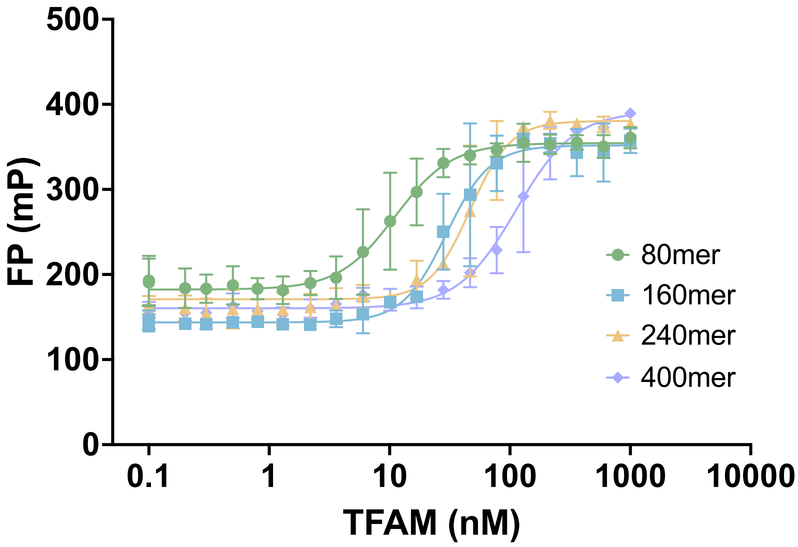

Length	EC_{50}	Hill slope
80mer	11 ± 2.7 nM	1.8
160mer	30 ± 5.1 nM	2.4
240mer	46 ± 13 nM	2.6
400mer	112 ± 21 nM	1.9

Figure S1. TFAM binds DNA cooperatively and with high affinity. Fluorescence Polarization (FP) analysis of TFAM with four DNA fragments of increasing length (randomized sequence). Data were fit to a four parameter Hill equation to obtain EC_{50} values and Hill slopes. n=2. Error shown in the inset was calculated from 95% confidence intervals.

bioRxiv preprint doi: https://doi.org/10.64898/2026.04.24.720727; this version posted April 27, 2026. The copyright holder for this preprint (which was not certified by peer review) is the author/funder, who has granted bioRxiv a license to display the preprint in perpetuity. It is made available under aCC-BY 4.0 International license.

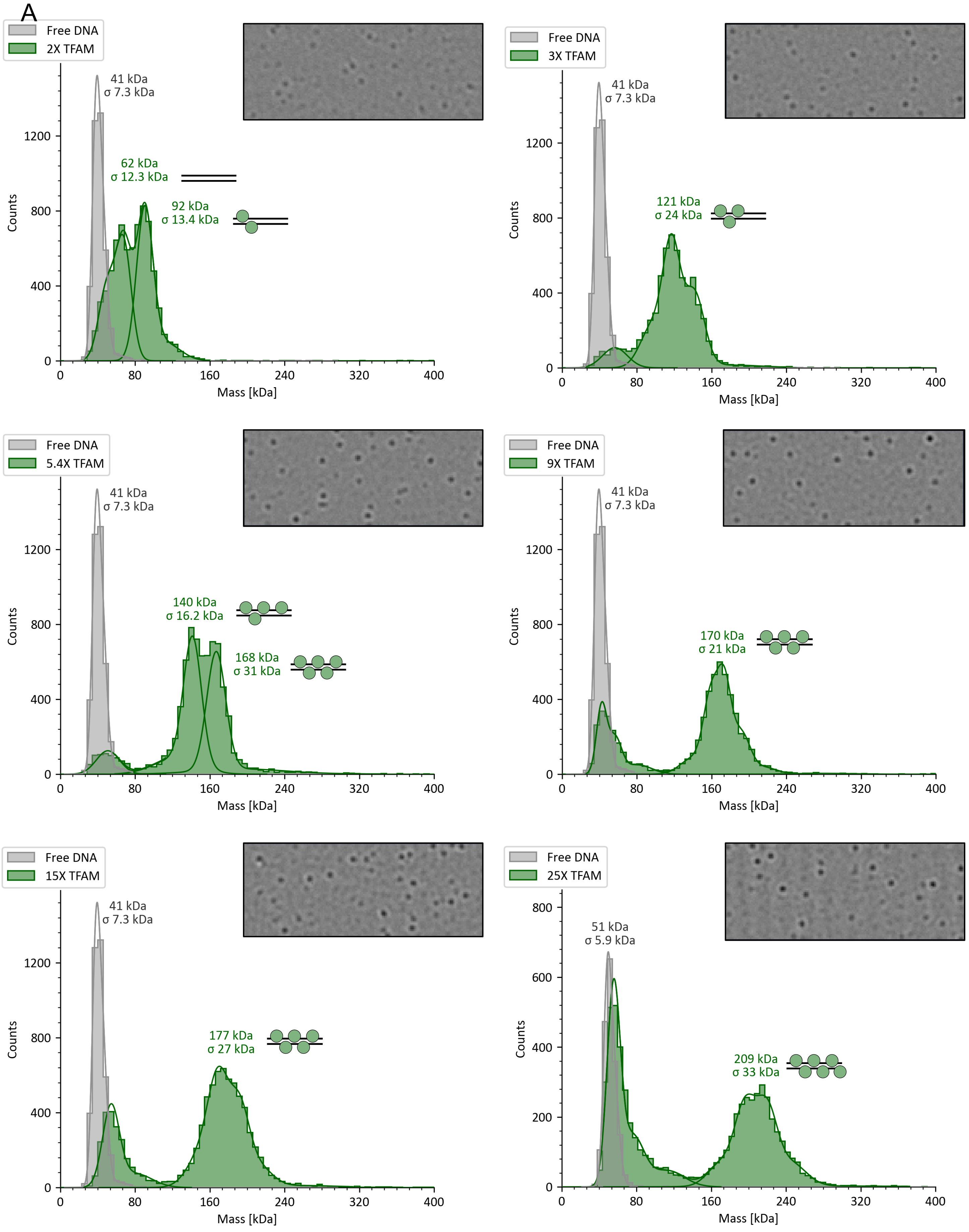

bioRxiv preprint doi: https://doi.org/10.64898/2026.04.24.720727; this version posted April 27, 2026. The copyright holder for this preprint (which was not certified by peer review) is the author/funder, who has granted bioRxiv a license to display the preprint in perpetuity. It is made available under aCC-BY 4.0 International license.

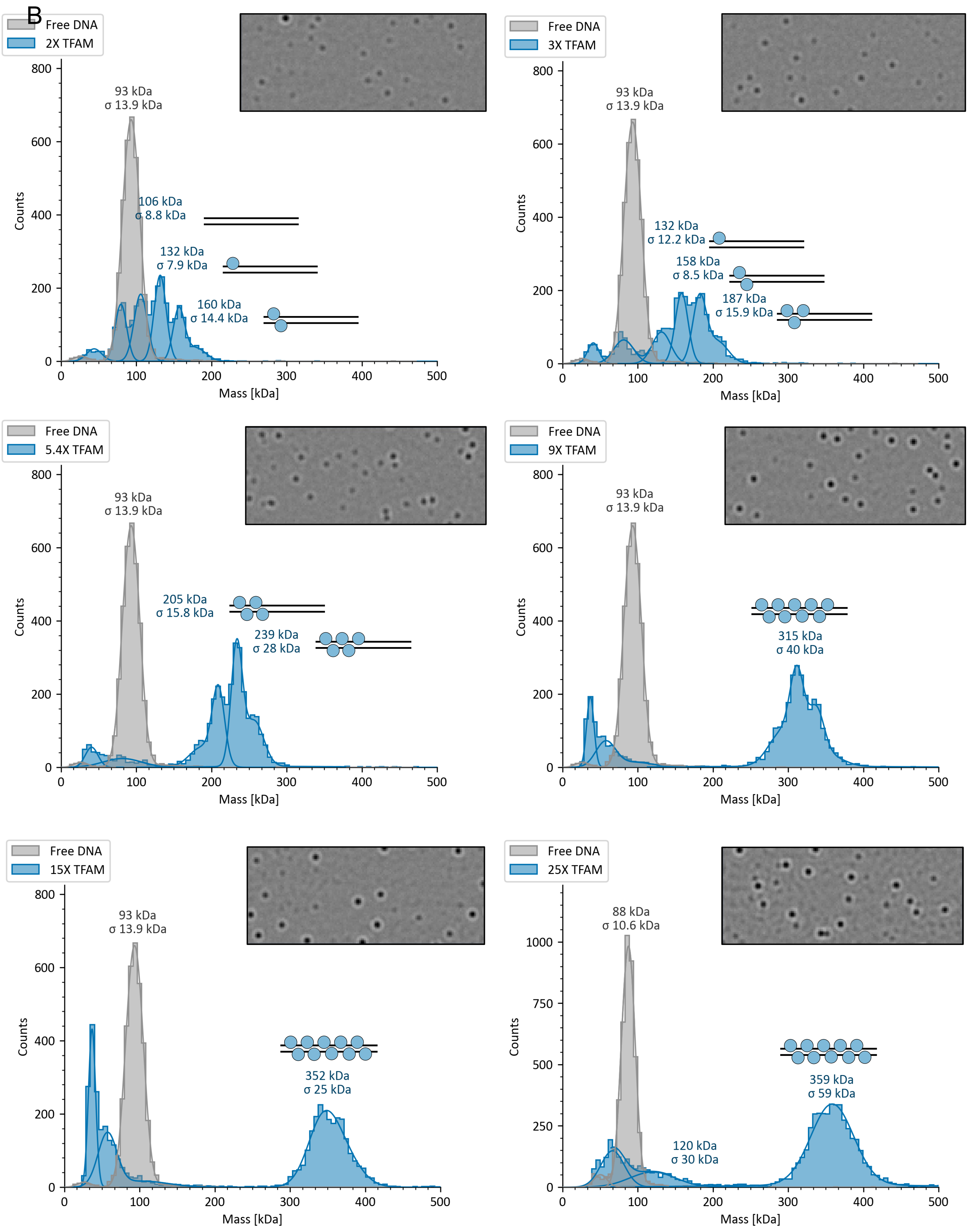

bioRxiv preprint doi: https://doi.org/10.64898/2026.04.24.720727; this version posted April 27, 2026. The copyright holder for this preprint (which was not certified by peer review) is the author/funder, who has granted bioRxiv a license to display the preprint in perpetuity. It is made available under aCC-BY 4.0 International license.

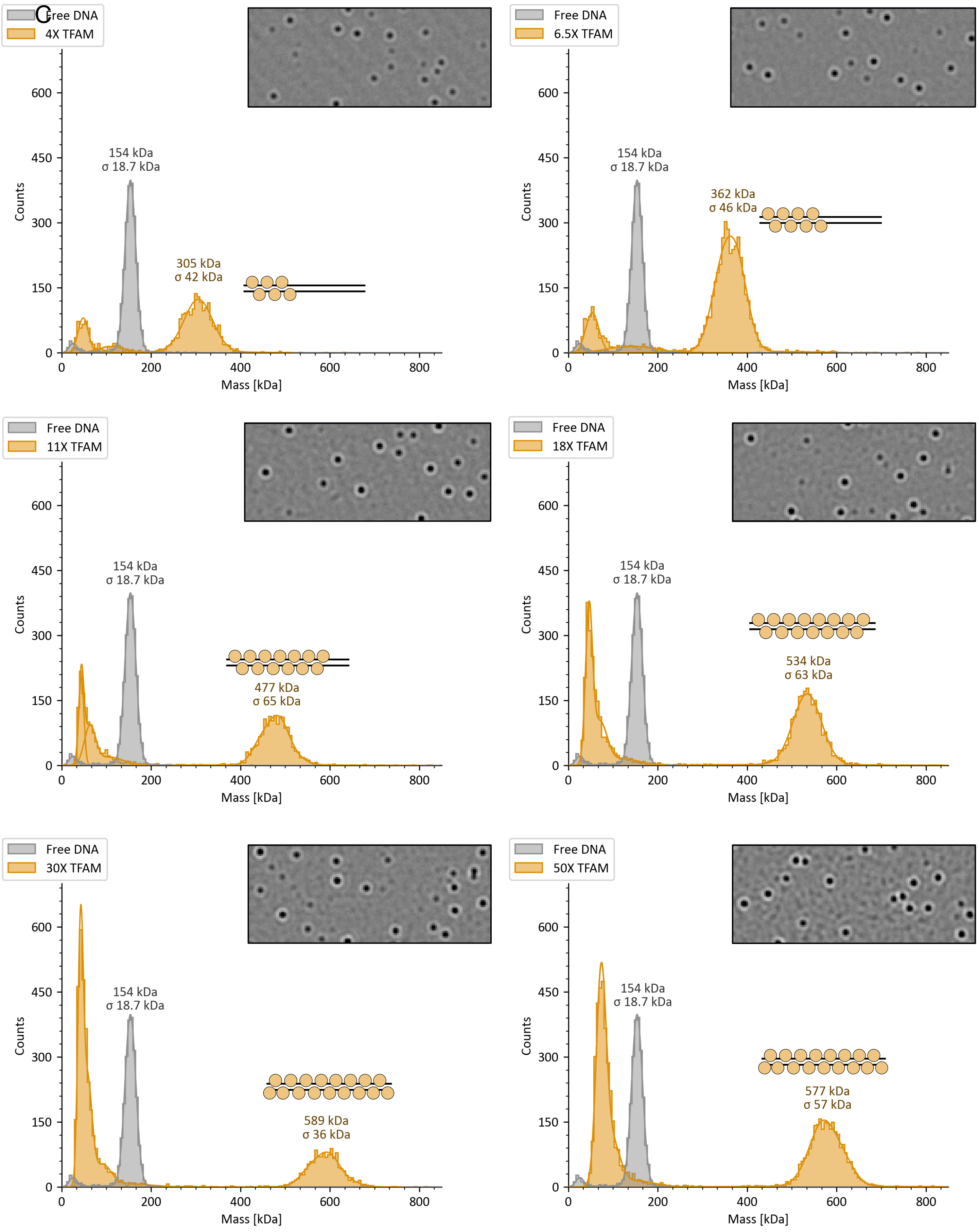

bioRxiv preprint doi: https://doi.org/10.64898/2026.04.24.720727; this version posted April 27, 2026. The copyright holder for this preprint (which was not certified by peer review) is the author/funder, who has granted bioRxiv a license to display the preprint in perpetuity. It is made available under aCC-BY 4.0 International license.

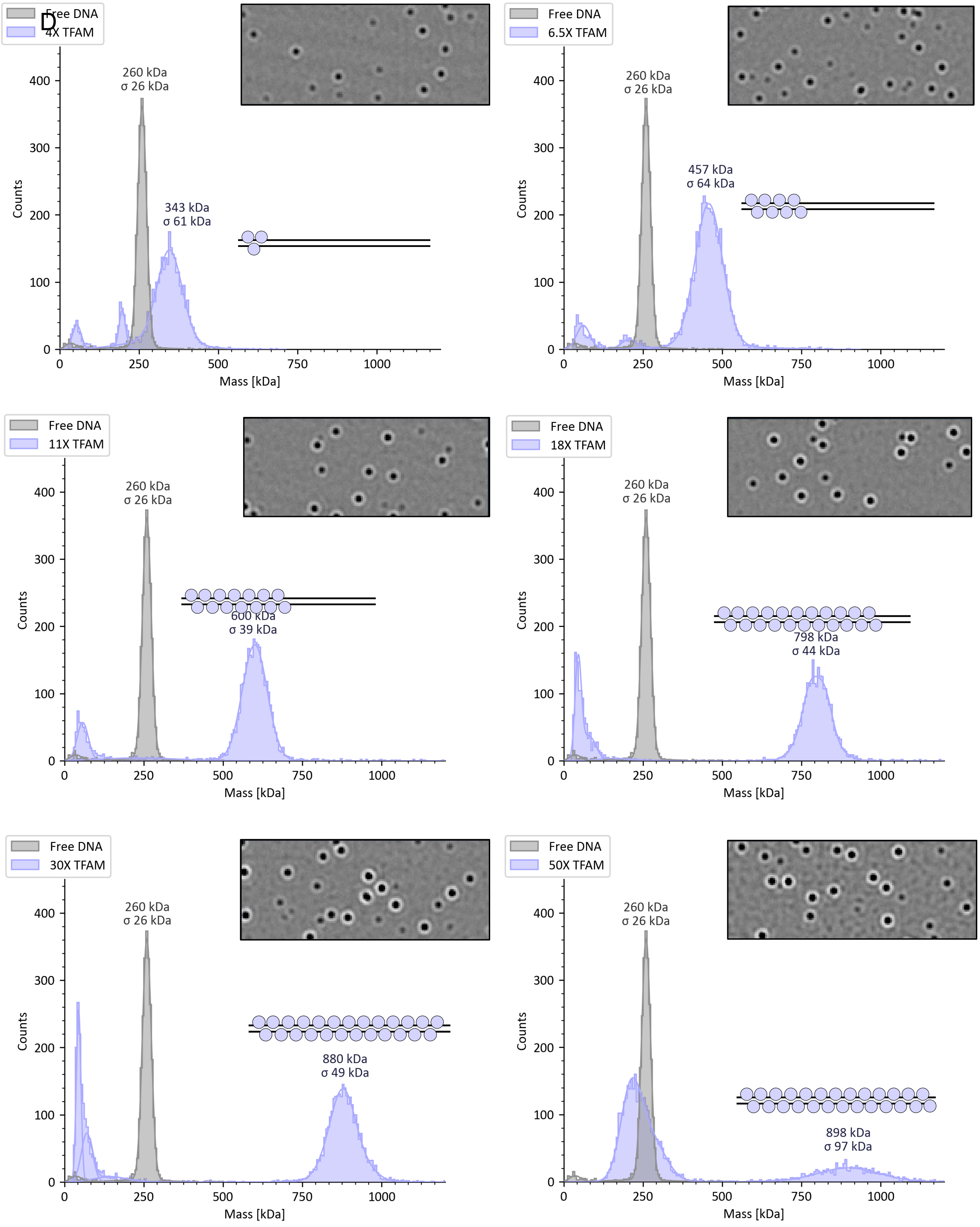

bioRxiv preprint doi: https://doi.org/10.64898/2026.04.24.720727; this version posted April 27, 2026. The copyright holder for this preprint (which was not certified by peer review) is the author/funder, who has granted bioRxiv a license to display the preprint in perpetuity. It is made available under aCC-BY 4.0 International license.

Figure S2. Mass photometry data for each titration point with DNA fragments of different lengths and with randomized sequences. A. TFAM titration into 80 bp DNA. **B.** TFAM titration into 160 bp DNA. **C.** TFAM titration into 240 bp DNA. **D.** TFAM titration into 400 bp DNA. Cartoon near each peak represents the number of TFAMs bound in each detected complex. Representative data shown.

bioRxiv preprint doi: https://doi.org/10.64898/2026.04.24.720727; this version posted April 27, 2026. The copyright holder for this preprint (which was not certified by peer review) is the author/funder, who has granted bioRxiv a license to display the preprint in perpetuity. It is made available under aCC-BY 4.0 International license.

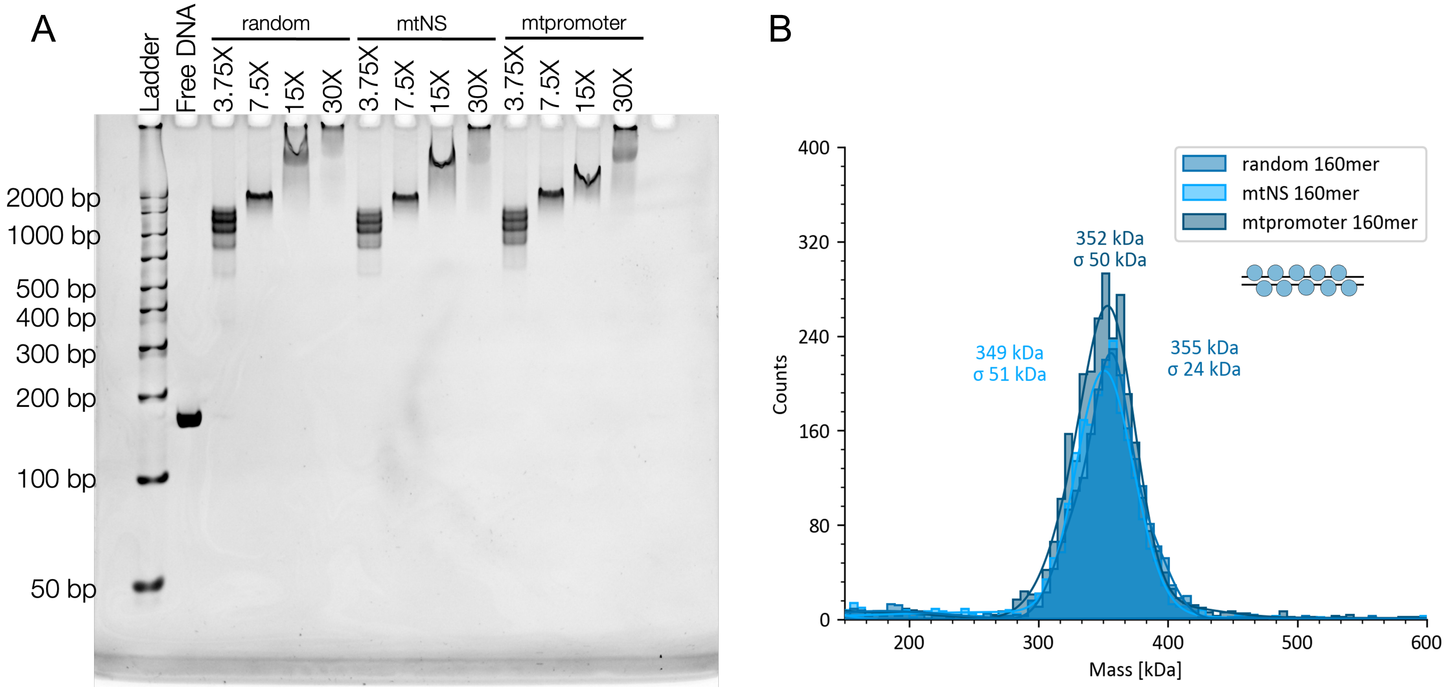

Figure S3. TFAM oligomerization is not sequence specific. A. EMSA with 160 bp fragments of different sequences: random DNA sequence, a nonspecific mtDNA sequence ("mtNS"), and a specific mtDNA sequence ("mtpromoter"). Samples were run on a 6% DNA retardation gel and visualized by ethidium bromide staining. Representative of n=2 shown. **B.** Mass photometry of the saturated complex (15X TFAM) with each DNA fragment. Representative of n=3 for random 160mer, and n=1 for mtNS and mtpromoter DNAs.

bioRxiv preprint doi: https://doi.org/10.64898/2026.04.24.720727; this version posted April 27, 2026. The copyright holder for this preprint (which was not certified by peer review) is the author/funder, who has granted bioRxiv a license to display the preprint in perpetuity. It is made available under aCC-BY 4.0 International license.

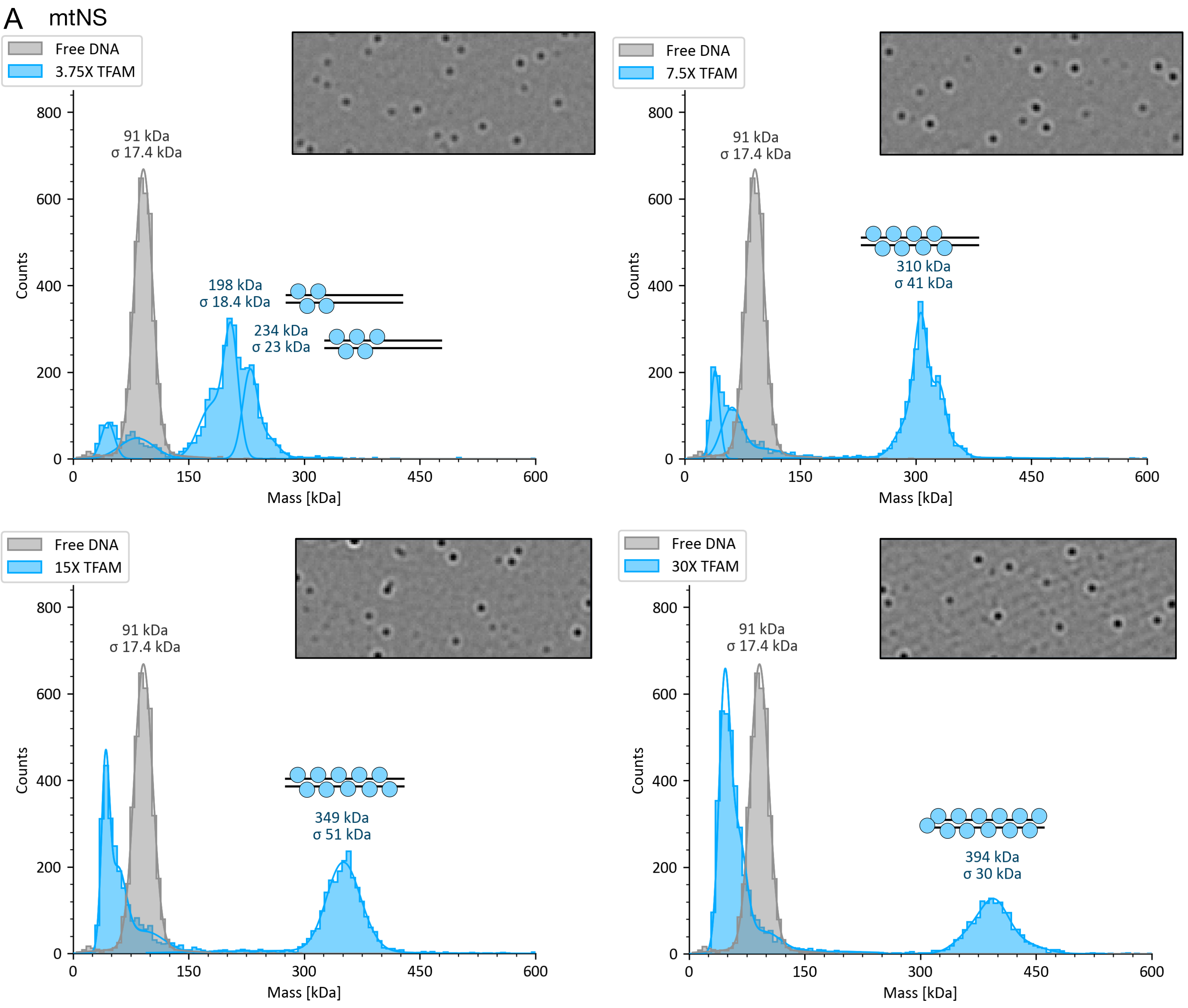

bioRxiv preprint doi: https://doi.org/10.64898/2026.04.24.720727; this version posted April 27, 2026. The copyright holder for this preprint (which was not certified by peer review) is the author/funder, who has granted bioRxiv a license to display the preprint in perpetuity. It is made available under aCC-BY 4.0 International license.

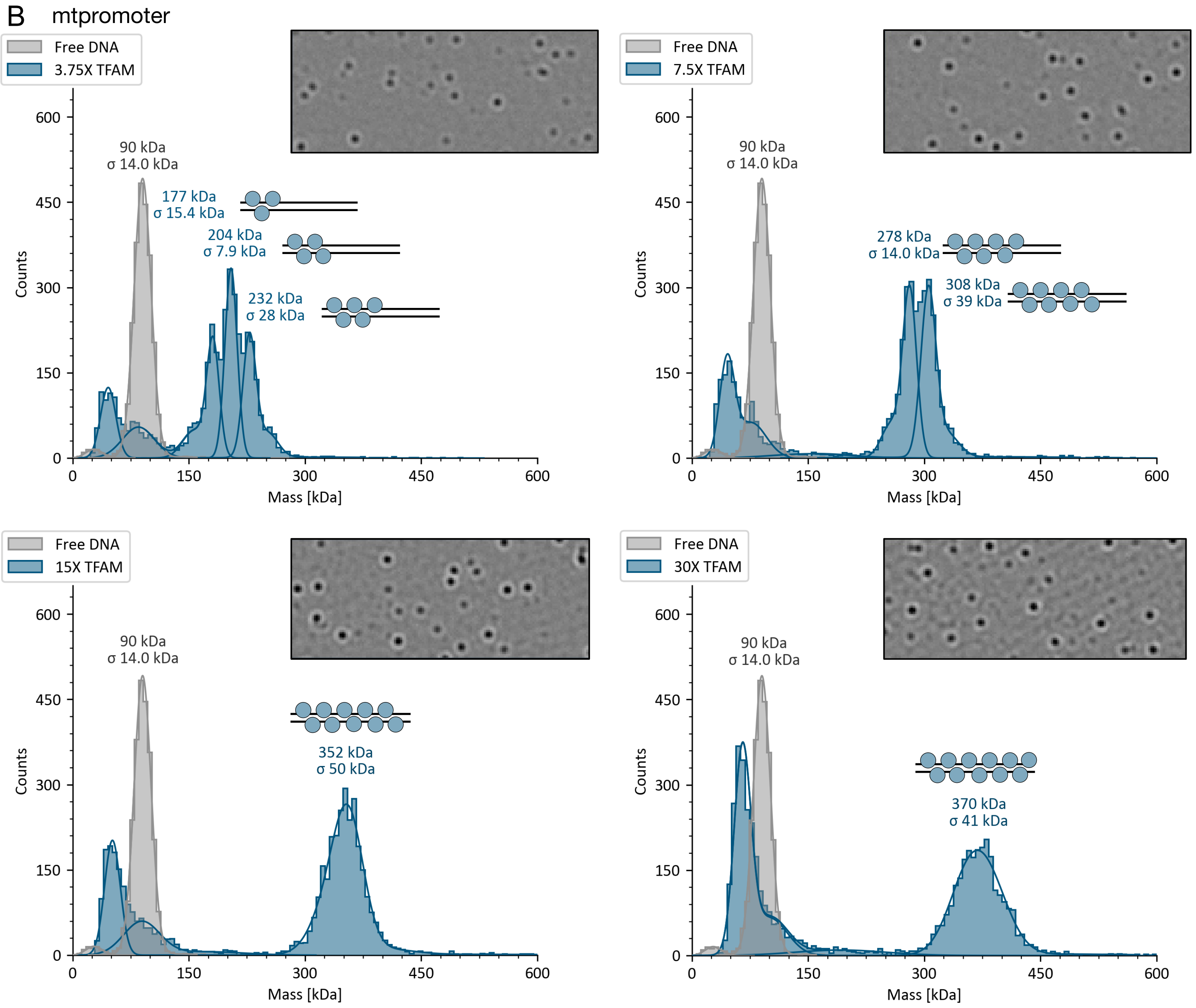

Figure S4. Mass photometry data for full titration with mitochondrial DNA-derived DNAs. A. TFAM titration into 160 bp mtNS DNA. **B.** TFAM titration into 160 bp mtpromoter DNAs. Cartoon near each peak represents the number of TFAMs bound in each detected complex.

bioRxiv preprint doi: https://doi.org/10.64898/2026.04.24.720727; this version posted April 27, 2026. The copyright holder for this preprint (which was not certified by peer review) is the author/funder, who has granted bioRxiv a license to display the preprint in perpetuity. It is made available under aCC-BY 4.0 International license.

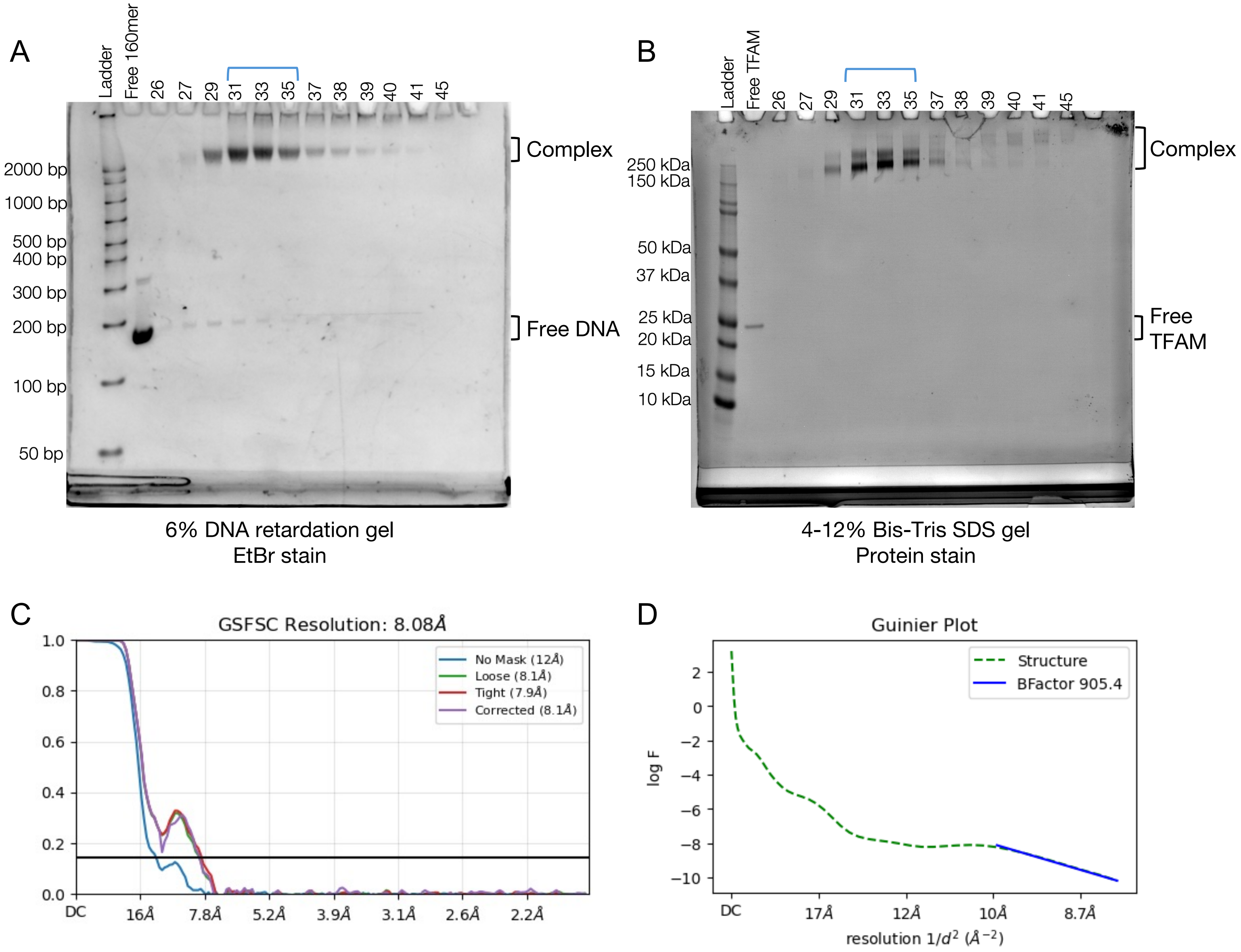

Figure S5. Sample preparation and refinement data for crosslinked TFAM-160mer complex. A. Fractions analyzed on 6% DNA retardation gels (Invitrogen) stained with Ethidium Bromide **B.** Fractions analyzed on SDS-PAGE gels stained with Blazin' blue. Pooled fractions are indicated by the blue bracket. **C.** Gold standard FSC curve for the density map from homogenous refinement. **D.** Guinier plot from homogenous refinement used to estimate B-factor of the density map.

bioRxiv preprint doi: https://doi.org/10.64898/2026.04.24.720727; this version posted April 27, 2026. The copyright holder for this preprint (which was not certified by peer review) is the author/funder, who has granted bioRxiv a license to display the preprint in perpetuity. It is made available under aCC-BY 4.0 International license.

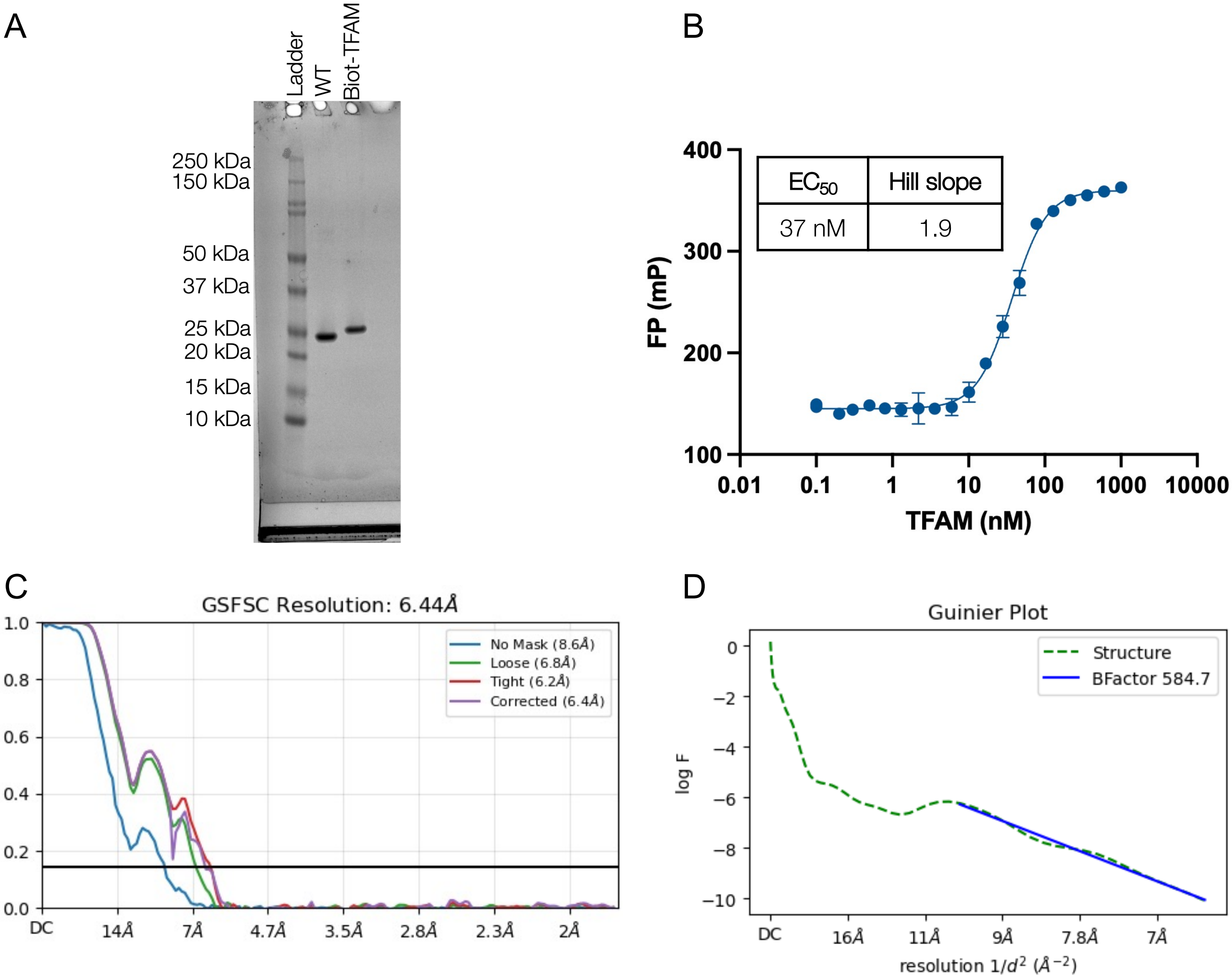

Figure S6. TFAM biotinylation for streptavidin monolayer affinity grids and refinement data for native TFAM-160mer complex. A. Verification of TFAM modification using 4-12% SDS-PAGE stained with Ethidium Bromide. **B.** Fluorescence Polarization with biotinylated TFAM and Alexa Fluor 488-labeled 160 bp DNA showing a similar binding affinity to wild type TFAM. **C.** Gold standard FSC curve for the density map from homogenous refinement. **D.** Guinier plot from homogenous refinement used to estimate B-factor of the density map.

bioRxiv preprint doi: https://doi.org/10.64898/2026.04.24.720727; this version posted April 27, 2026. The copyright holder for this preprint (which was not certified by peer review) is the author/funder, who has granted bioRxiv a license to display the preprint in perpetuity. It is made available under aCC-BY 4.0 International license.

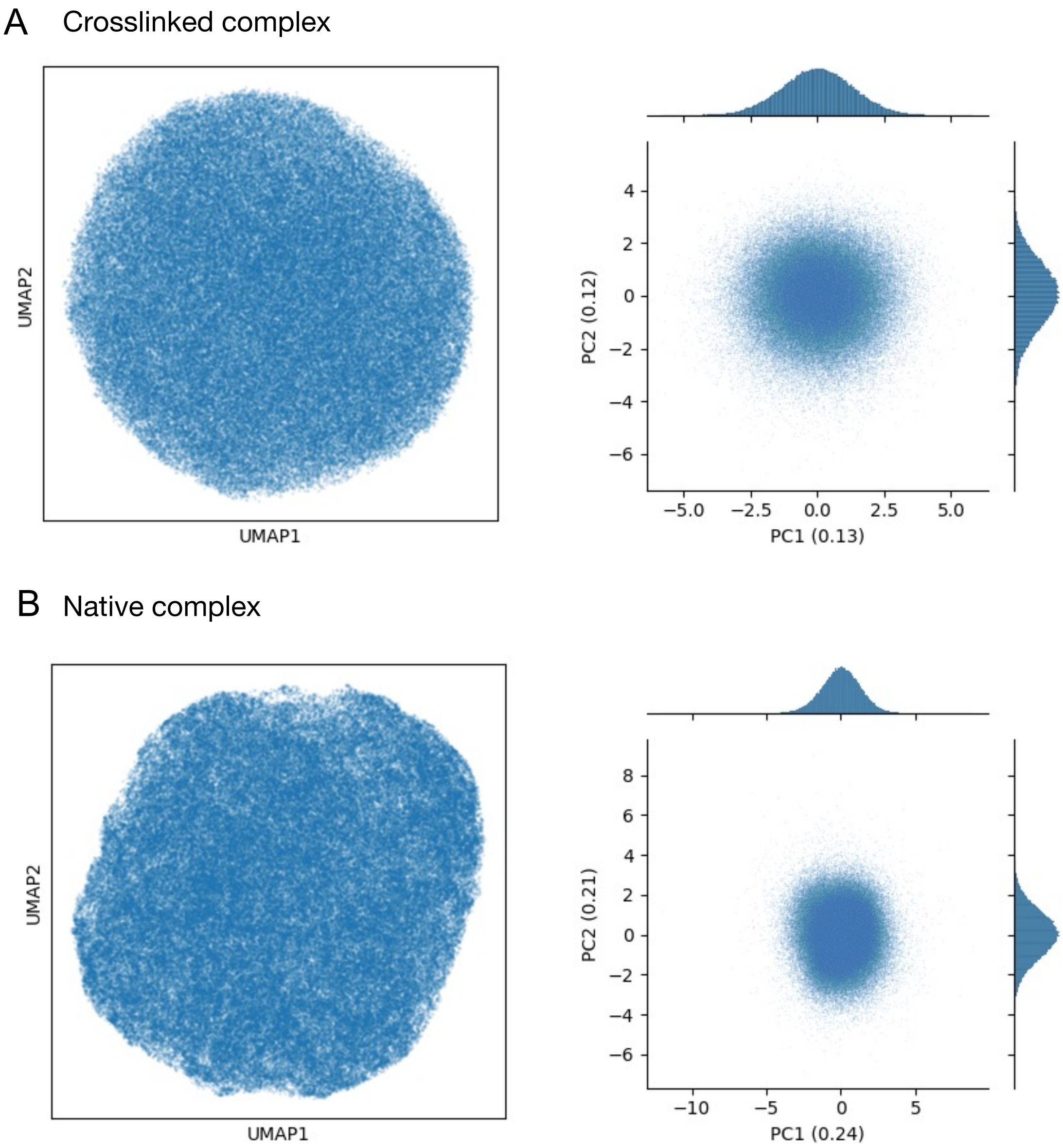

Figure S7. cryoDRGN analysis of native and crosslinked TFAM-160mer complexes. A. UMAP visualization (left) of the latent variable distribution and Principal Component Analysis (PCA) projection (right) of latent space encodings after training on a 10-dimensional latent variable model in cryoDRGN for the crosslinked complex. **B.** UMAP visualization (left) of the latent variable distribution and Principal Component Analysis (PCA) projection (right) of latent space encodings after training on a 10-dimensional latent variable model in cryoDRGN for the native complex.
